# Exposure to Bisphenol S and Bisphenol F Alters Gene Networks Related to Protein Translation and Neuroinflammation in SH-SY5Y Human Neuroblastoma Cells

**DOI:** 10.3390/toxics13090772

**Published:** 2025-09-12

**Authors:** Andrea P. Guzman, Christina L. Sanchez, Emma Ivantsova, Jacqueline Watkins, Sara E. Sutton, Christopher L. Souders, Christopher J. Martyniuk

**Affiliations:** Interdisciplinary Program in Biomedical Sciences Neuroscience, Center for Environmental and Human Toxicology, Department of Physiological Sciences, College of Veterinary Medicine, UF Genetics Institute, University of Florida, Gainesville, FL 32611, USA; apguzman525@gmail.com (A.P.G.); chrissylaurasanchez@gmail.com (C.L.S.); eivantsova@ufl.edu (E.I.); jmecalwatkins@ufl.edu (J.W.); sarasutton@ufl.edu (S.E.S.); ksouders@ufl.edu (C.L.S.II)

**Keywords:** bisphenol replacements, neuro-immune interactions, RNA-seq, xenobiotics, caspase activity, acute toxicity tests

## Abstract

Bisphenol A (BPA) replacement chemicals are used in products like food packaging, plastic piping, and sportswear. While they can be toxic, their neurotoxicity is less understood. The aim of this study was to treat differentiated human SH-SY5Y cells with Bisphenol S (BPS) and Bisphenol F (BPF) to investigate mechanisms of toxicity. BPS reduced cell viability (>50 µM at 48 h) more than BPF (>200 µM at 48 h), with concentration- and time-dependent effects. Both induced caspase 3/7 activity at 250 µM after 48 h, though no changes were observed in levels of reactive oxygen species nor mitochondrial ATPase activity. RNA-seq analysis at 0.1 nM revealed distinct transcriptional networks: BPS altered IL15R, causing NF-kB/NFATC activation, and triggered NF-kB signaling through CD8, while BPF affected TLR9 and activated NF-kB targets through TNF. Pathway analysis showed that genes involved in neuroinflammation, protein folding, microglial function, and motor neuron regulation were disrupted, demonstrating that BPS and BPF, even at low, environmentally relevant concentrations, significantly alter gene expression in pathways linked to neuroinflammation, immune signaling, and neurodegenerative diseases. BPS primarily affected ribosomal and immune-related networks, while BPF disrupted oxidative phosphorylation and protein-folding pathways. These alterations suggest mechanisms for long-term neurological effects, highlighting the need for comprehensive evaluations of BPA alternatives.

## 1. Introduction

Polycarbonate plastics containing Bisphenol A (BPA) were once widely used in manufactured products such as babies’ and children’s plastic cups, water bottles, plastic wraps, pre-packaged food containers, and sealants of the inner lining of metal cans; however, there is strong evidence of widespread BPA-induced reproductive dysfunction in wildlife (i.e., fish species [1]) and that exposures can lead to impairment of both the male [2] and female [3,4] mammalian reproductive system. The reproductive toxicity of BPA involves various mechanisms including estrogen receptor (ER) activation, binding to membrane bound G-protein coupled receptors (GPCRs) [5] and competitive inhibition of androgen receptors [6], as well as other steroid receptor-related mechanisms. In addition to reproduction, other physiological systems are disrupted following BPA exposure, either through direct effects via estrogen receptor activation or through other receptors involving the immune system, metabolism, and behavior [7,8]. There are also wildlife and human health concerns for prolonged exposure to BPA, as it has been quantified in human urine, human tissues, and tissues of wildlife [9]. Consequently, BPA has gradually been phased out of many products and is now under strict environmental- and human-health-related regulations for safety.

Due to the restrictions and/or bans of BPA usage in several countries, bisphenol replacements are used extensively in plastic products. These “BPA alternatives” are those primarily containing two hydroxyphenyl functionalities (i.e., bisphenol B, F, G, S, P, Z). BPF, for example, is useful in epoxy coatings needed for flooring, linings for storage tanks, road coverings, industrial adhesives and grouts, water pipes, and electrical varnishes [10]. Both BPF and BPS have also been measured in consumers’ products like personal care products, paper products, and food [10,11]. In the environment, bisphenols replacements are detectable in surface water, sewage, and sediment like BPA. Environmental concentrations of BPS and BPF are typically reported at low levels (ng/L) [12]; however, they can reach over 1 µg/L at highly contaminated sites [13,14]. Similarly, studies performed on human fluids and tissue report low levels (ng/L) of these compounds [15,16], but they also can be reported at higher levels in varying exposure groups [17]. These analogs share a common chemical structure to BPA and many may bind human ERs with greater affinity than BPA [18]. Thus, replacement products for BPA continue to be under scientific scrutiny for their role in potential disruptions to the reproductive system. There is evidence that bisphenol replacements impair not only reproduction in mammals [19], but also immune function [20], cardiovascular function [21], and metabolism [22].

Recently, concerns have been raised regarding the exposure effects of BPA replacement chemicals in relation to neurodevelopment and neurotoxicity of wildlife and humans. Studies have detected BPA and BPF in the human brain at concentrations up to 2.4 ng/g [23] and these chemicals have been associated with impaired early neural development in children [24,25]. Other adverse neurological effects include dose-dependent delays or impairment of neuron and oligodendrocyte maturation with exposure to BPA and BPF [26]. Recent studies on BPA, BPB, and BPS in neuronal cell lines show that there can be neurotoxic effects involving reactive oxygen species (ROS), leading to increased malondialdehyde (MDA) content [27]. Experimental evidence for oxidative stress and neurotoxicity has also been observed in non-mammalian animal models like zebrafish [28,29,30].

The aim of the present study was to address knowledge gaps in how bisphenol replacements may affect the brain by improving our understanding of their neurotoxicity in neuronal cells; thus, we exposed human SH-SY5Y neuroblastoma cells to BPS and BPF. Human SH-SY5Y neuroblastoma cells are valuable in in vitro models not only for their reliability in studying neuronal function and neurotoxicity, but also for the development of more physiologically relevant data compared to animal-derived cell lines. Additionally, we chose to focus on BPS and BPF, as these replacements are increasingly being utilized despite their full toxicity mechanisms remaining understudied. We hypothesized that both bisphenol replacements would induce neurotoxicity via (1) neuroinflammation and/or (2) oxidative stress, leading to disruption in mitochondrial membrane potential and the oxidative phosphorylation of SH-SY5Y cells. We also quantified the molecular response in cells using RNA-seq to elucidate mechanisms underlying any observed neurotoxicity at low concentrations to reflect concentrations in the human brain.

## 2. Materials and Methods

### 2.1. Chemicals

PESTANAL^®^, analytical standards for both BPF (4,4′-Methylenediphenol) (CAS Number 620-92-8) and BPS (4,4′-Sulfonyldiphenol) (CAS Number 80-09-1) were purchased from Sigma-Aldrich (St. Louis, MO, USA). Nominal stock solutions of BPS and BPF were prepared by dilution in 0.1% dimethyl sulfoxide (DMSO) (CAS no. 67-68-5, purity ≥99.9%, Sigma-Aldrich). Oligomycin A (≥99%, CAS Number 579-13-5), carbonyl cyanide-4-phenylhydrazone (FCCP) (≥98%, CAS Number: 370-86-5), and antimycin A (≥95%, CAS Number 1397-94-0) were also purchased from Sigma for mitochondrial assays.

### 2.2. SH-SY5Y Culturing, Differentiation, and Chemical Exposures

Cell culturing of the differentiated SH-SY5Y cells have been outlined previously in our publication [31]. The complete method is provided in Supplemental Methods. Cells were purchased from ATCC^®^ CRL 2266™ (ATCC, Manassas, VA, USA) and validated by the company through STR profiling. To produce a more neuronal phenotype, SH-SY5Y cells were exposed to retinoic acid (RA) (10 μM) for 6 days as per established methods [32,33]. Details on media are given in Supplemental Methods. Differentiated SH-SY5Y cells were maintained in DMEM:F12 with L-glutamine media supplemented with 1% (*v*/*v*) fetal bovine serum, and 1% Antibiotic-Antimycotic solution (Gibco) in a humidified atmosphere of 5% CO_2_ at 37 °C [34].

### 2.3. Cell Viability Assay

The CellTiter-Glo^®^ Luminescent Cell Viability Assay (Promega, Madison, WI, USA) was used to determine the proportion of viable SH-SY5Y cells in the culture. SH-SY5Y cells were exposed to media only, 0.1% DMSO solvent control, positive control (20 µM Antimycin/Rotenone), or one concentration of either BPF or BPS at 0.1, 0.5, 1, 5, 10, 50, 100, 150, 200, or 250 µM (*n* = 4). The luminescence was recorded using a Synergy™ 4 Hybrid Microplate Reader (BioTek, Winooski, VT, USA).

### 2.4. Caspase Activity Assay

SH-SY5Y cells were collected and seeded at *n* = 3 (wells/concentration) after being differentiated for 6 days with retinoic acid. BFS or BPF was added over a concentration range of 0.1–250 µM. Approximately 100 µL of media containing ~10,000 cells were seeded in each well of a 96-well plate. Positive controls included 30 µg/mL digitonin (DIG), and either 1, 10, or 100 µM antimycin A and rotenone at each time point. These chemicals induce cytotoxicity and caspase activity. Each time point was conducted on different plates. At either 4, 24, and 48 h, 100 µL of Caspase-Glo 3/7 reagent (Promega) was added to each well. After 30 s of orbital shaking at 400 rpm, the plate was incubated for 1.5 h at room temperature. A Synergy™ 4 Hybrid Microplate Reader (BioTek) was used to measure luminescence. Background fluorescence of cells was considered and controlled for each assay (background corrected).

### 2.5. Mitochondrial Membrane Potential

Cell culturing of the differentiated SH-SY5Y cells has been outlined previously in our publication [31]. The complete method is provided in Supplemental Methods. Mitochondrial membrane potential (MMP) was determined by the Mitochondrial Membrane Potential Kit (Sigma-Aldrich). Cells were exposed to either media only, 0.1% DMSO solvent control, positive control (4 and 8 μM FCCP), or one concentration of either BPF or BPS at 0.001, 0.01, 0.1, 0.5, 1, 5, 10, 50, 100, 150, 175, 200, and 250 μM (*n* = 3). A Synergy™ 4 Hybrid Microplate Reader (BioTek) was used to measure luminescence. The experiment was conducted twice, independently.

### 2.6. ATPase Activity

Cell culturing of the differentiated SH-SY5Y cells has been outlined previously in our publication [35]. The complete method is provided in Supplemental Methods. Cells were exposed in quadruplicate for 24 h to one concentration of 10, 50, 100, 150, 175, or 250 µM BPS or BPF, or the positive control (10 µM oligomycin, OM) (ATPase Assay Kit, ab234055). A BioTek Synergy H1 Spectrophotometer was used for a kinetic assay.

### 2.7. Mitochondrial Bioenergetics in Differentiated SH-SY5Y Cells

Cell culturing of the differentiated SH-SY5Y cells has been outlined previously in our publication [31]. The complete method is provided in Supplemental Methods. Mitochondrial bioenergetics was analyzed using a Seahorse XFe24 flux analyzer (Agilent Technologies, Santa Clara, CA, USA) following the standard mitochondrial stress test regimen. Cells were exposed to media only, 0.1% DMSO, or one final concentration of either 0.1 nM, 0.1 μM, 100 μM, or 250 μM BPS, using direct injection.

### 2.8. Intracellular Reactive Oxygen Species in Differentiated SH-SY5Y Cells

The quantification of reactive oxygen species in differentiated SH-SY5Y cells have been described previously in our publications [31,35]. The full methods are provided in Supplemental Methods. ROS production was measured using (1) the ROS-Glo™ H_2_O_2_ Assay (Promega, Cat #G8820) as per the manufacturer’s protocol and (2) with the fluorescent probe DCFDA (2′,7′-dichlorofluorescein diacetate). Two assays were employed to capture different ROS dynamics. ROS-Glo™ (Promega, Madison, WI, USA) measured hydrogen peroxide and DCFDA measured general ROS production (via oxidation to a fluorescent product). Both assays had several negative and positive controls (i.e., established ROS inducers). Conducting two complimentary assays strengthens the rigor of the endpoint assessment.

### 2.9. RNA-Sequencing

All methods are provided in detail in the Supplemental Methods. Briefly, SH-SY5Y cells were exposed in T75 flasks by removing all the media and replacing it with 8 mL of differentiation media (DMEM:F12 containing 1% FBS, 1% antibiotic/antimycotic solution, and 10 µM retinoic acid) every 48 h. On the 6th day, cells were exposed to media, 0.1 nM BPS, or 0.1 nM BPF (*n* = 4) for 48 h. Cells were spun down and added into TRIzol™ Reagent (Thermo Fisher Scientific, Waltham, MA, USA) for total RNA extraction. Quality of samples was assessed using both the Qubit^®^ 2.0 Fluorometer (ThermoFisher, Grand Island, NY, USA) and Agilent 2100 Bioanalyzer (Agilent Technologies, Santa Clara, CA, USA). Twelve samples were used for RNA-seq library construction (RINs > 7).

Libraries and sequencing were conducted by Novogene (Novogene Corporation, Beijing, China) using the NEBNext Poly(A) mRNA Magnetic Isolation module (New England Biolabs, Ipswich, MA, USA, catalog # E7490). RNA library construction followed instructions outlined in the NEBNext^®^ Ultra™ II Directional RNA Library Prep Kit for Illumina^®^ (New England Biolabs, catalog #E7760). Individually prepared libraries were pooled by equimolar concentrations and sequenced using a NovoSeq 6000 instrument (150 bp paired end reads) (Illumina Inc., San Diego, CA, USA). Data were processed as outlined in our published manuscript [36]. The reference genome (homo_sapiens_grch38_p12_gca_000001405_27) was used for alignment of RNA-seq data. Differentially expressed transcripts were revealed as stated in Supplemental Methods.

### 2.10. Bioinformatics of Pathways

As a first-level analysis, enrichment analysis of the differential expressed genes was conducted for each concentration of BPF and BPS using the Gene Ontology (GO). Gene set enrichment analysis (GSEA) and subnetwork enrichment analysis (SNEA) were further conducted in Pathway Studio v12.0 (Elsevier, Amsterdam, The Netherlands). Details are found in Supplemental Methods as well as in previously published studies [36,37]. All raw and processed transcriptome data are available via the NCBI Gene Expression Omnibus (GEO) database (GSE217951, release date April 2024).

### 2.11. Real-Time PCR Analysis

Real-time PCR followed our established methods using TRIzol^®^ Reagent (Life Technologies, Carlsbad, CA, USA) [38]. Genomic DNA was removed using the TURBO DNA free™ Kit (Thermo Fisher Scientific). The cDNA was generated with the iScript™ Select cDNA Synthesis Kit (Bio-Rad, Hercules, CA, USA). For quantitation, the CFX Connect System (BioRad, USA) was used with SSo-Fast™ EvaGreen^®^ Supermix Kit (BioRad, Hercules, CA, USA). Samples were run in duplicate and cycling parameters have been previously described by us [38]. Further details are presented in Supplemental Methods.

The primers used in this study were obtained from the published literature or designed using NCBI Primer. Based on the functional enrichment, genes related to transcription/translation were measured. These included general transcription factor IIH subunit 4 (*Gtf2h4*) (BT007321.1) [F’ TATTGGACCGATTGTATGGGCA, R’ AGCCCTGTACTTTCCTCCTGA] Secretogranin V (*Scg5*) (NG_051230.1) [F’ CTGTCCTGTTGGAAAAACAGCAG, R’ GACACTCCTCCGCTTTCGTC], and Valyl-tRNA synthetase 2 (*V*ars*2*) (BC113605) [F’ ACAGCCCCCGATATGTTGAG, R’ GGCCTGATATTCTGGTTTGAAGA]. Expression data were normalized to *Gapdh* [F’ TGCACCACCAACTGCTTAGC, R’ GGCATGGACTGTGGTCATGAG] [39] using CFX Manager™ software (v3.1) (baseline subtracted) and the Cq method was employed.

### 2.12. Statistical Analysis

All statistical analyses were conducted in GraphPad Prism (La Jolla, CA, USA, version 9.4). Differences for cell viability data, caspase activation, MMP, ATP synthase levels and ROS were analyzed using a One-way ANOVA, followed by Dunnett’s multiple comparison test to the DMSO solvent control. Relative mRNA levels were first log transformed (log10) and then analyzed with ANOVA followed by a Dunnett’s test. Data is presented as mean ± S.D. unless otherwise stated in figure caption. Significance of difference was considered when *p* < 0.05 for all endpoints.

## 3. Results

### 3.1. Cytotoxicity Based on Cell Viability

**Figure 1:**
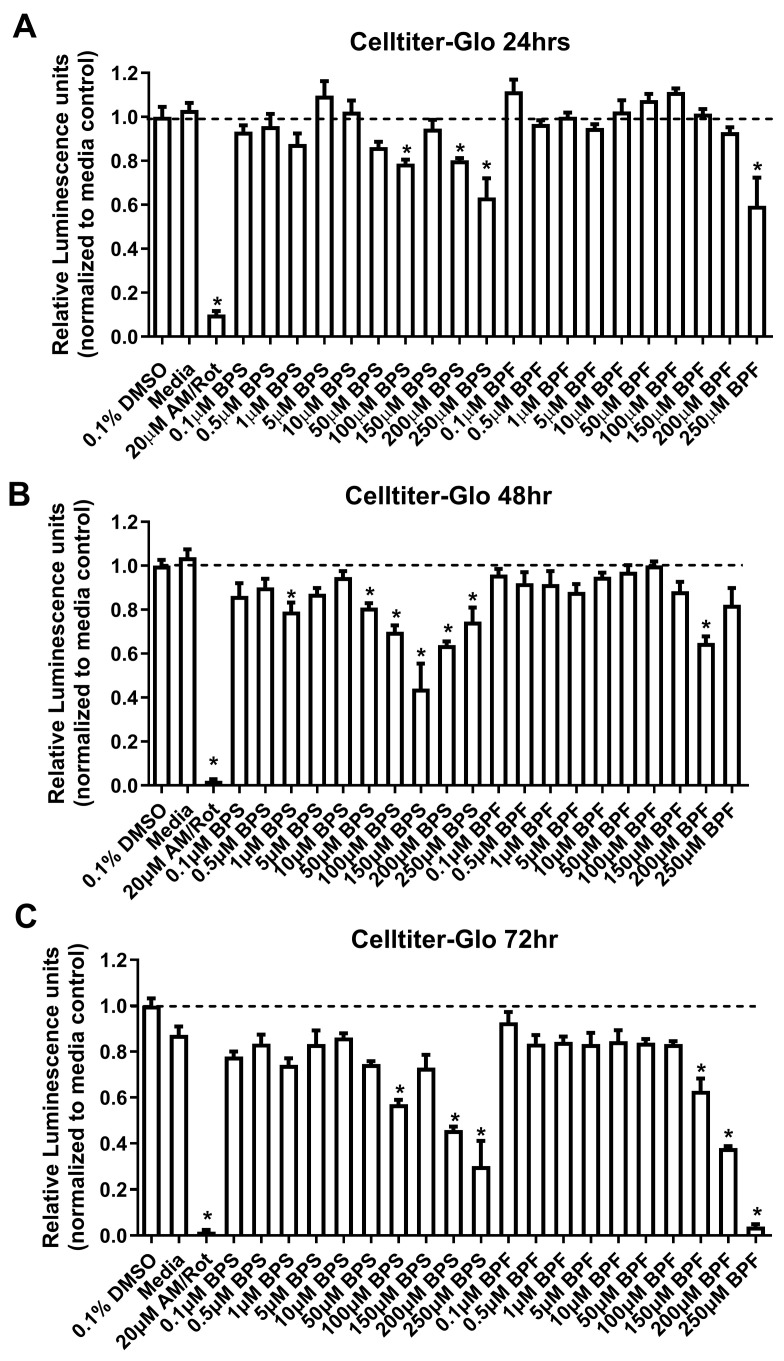
SH-SY5Y cell viability at (**A**) 24 h, (**B**) 48 h, or (**C**) 72 h following exposure to 0.1% DMSO, media alone, Antimycin/Rotenone (AM/Rot), or one concentration of either BPS or BPF at 0.1, 0.5, 1, 5, 10, 50, 100, 150, 200, or 250 µM. The column represents mean relative luminescence ± standard deviation relative to the DMSO control (One-way ANOVA with Dunnett’s multiple comparison test, *n* = 4/experiment). The hatched line represents 100% survival of cells for the DMSO treatment. Any signal above the line is 100% survival of cells. Asterisks indicate significant differences at * *p <* 0.05.

For all time points, the positive control (antimycin/rotenone, AM/Rot) reduced cell viability (Figure 1). At 24 h, there were significant effects on cell viability [F _(DFn, DFd)_, *p* value] (F _(22, 62)_ = 25.8, *p* < 0.0001) (Figure 1A). When comparing groups to the DMSO control, BPS reduced cell viability at 100 µM and above while BPF reduced cell viability at 250 µM. At 48 h, there were significant effects on cell viability (F _(22, 70)_ = 24.59, *p* < 0.0001) (Figure 1B). When comparing groups to the DMSO control, BPS reduced cell viability at ~50 µM and above while BPF reduced cell viability at 200 µM. At 72 h, there were significant effects on cell viability (F _(22, 69)_ = 44.19, *p* < 0.0001) (Figure 1C). BPS reduced cell viability at 100 µM and above while BPF reduced cell viability at 150 µM. Taken together, BPS exerted higher toxicity more rapidly to differentiated SH-SY5Y cells, relative to BPF.

### 3.2. Caspase 3/7 Activity

**Figure 2:**
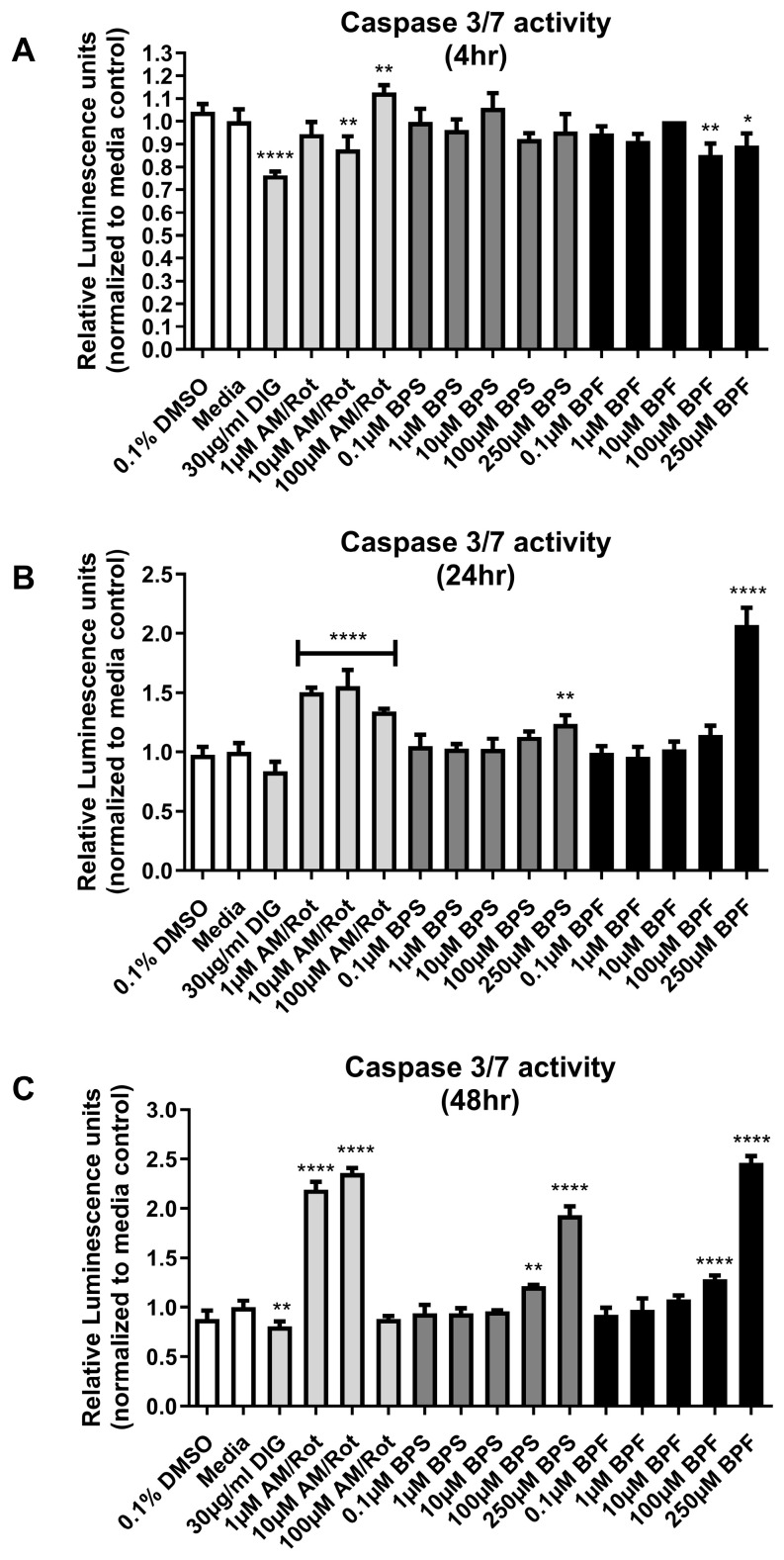
Caspase 3/7 activity after (**A**) 4 h, (**B**) 24 h, and (**C**) 48 h exposure to 0.1% DMSO, media alone, digitonin (DIG), Antimycin/Rotenone (1, 10, or 100 µM AM/Rot), or one concentration of either BPF or BPS at 0.1, 1, 10, 100, or 250 µM. The column represents mean relative luminescence ± standard deviation from the media control. Shading depicts the following groups (white = negative controls; light gray = positive controls; dark gray = BPS; black = BPF) (One-way ANOVA with Dunnett’s multiple comparison test, *n* = 3/experiment). Asterisks indicate significant differences at * *p <* 0.05, ** *p* < 0.01, **** *p* < 0.0001.

For Casp3/7-mediated apoptosis, positive controls for the assay included 1, 10, 100 µM antimycin/rotenone and cells treated with 30 µg/mL DIG. At 4 h, the positive control of 10 and 100 µM AM/Rot differed from the control (F _(15,38)_ = 13.31, *p* < 0.0001) (Figure 2A). BPF reduced Casp3/7 slightly, which may be related to cell viability at the higher concentrations. At 24 h, the positive controls for AM/Rot induced casp3/7 activity, as well as the 250 µM of both BPS and BPF (F _(15,38)_ = 50.43, *p* < 0.0001) (Figure 2B). At 48 h, the positive control of 1 µM and 10 µM antimycin/rotenone, as well as the 100 and 250 µM BPF and BPS differed significantly from the control (F _(15,38)_ = 280.9, *p* < 0.0001) (Figure 2C). The reduction in casp3/7 activity with the stronger positive controls at this later time point reflects the loss of cell viability in the assay due to toxicity.

### 3.3. Mitochondrial Membrane Potential

**Figure 3:**
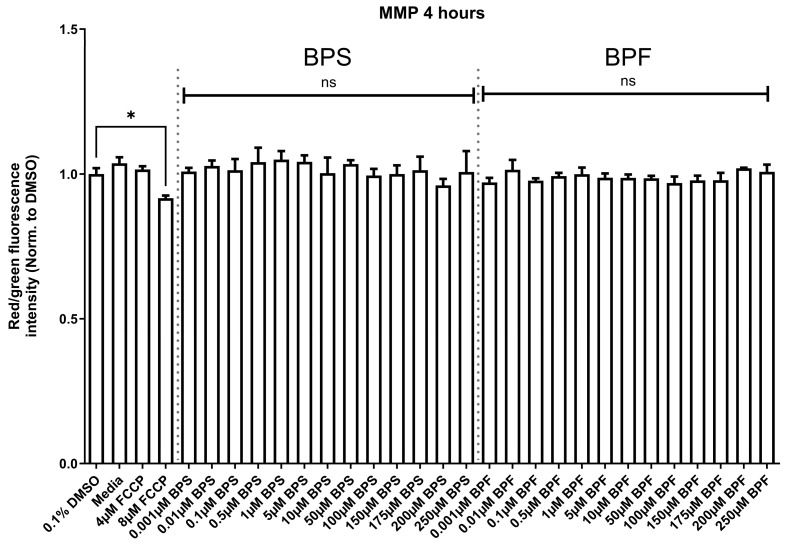
Mitochondrial membrane potential (MMP) of SH-SY5Y cells treated with 0.1% DMSO, media alone, 4 and 8 μM carbonyl cyanide-4-phenylhydrazone (FCCP), or one concentration of either BPF or BPS at 0.001 µM up to 250 µM after 4 h. The experiment was conducted twice with the same results (Supplemental Figure S1 shows the second experiment). Data are expressed as relative mean intensity units ± standard deviation standardized to the DMSO solvent control (One-way ANOVA followed by a Dunnett’s multiple comparison test, *n* = 3/treatment/experiment). Each treatment was compared to the DMSO control. Asterisks indicate significant differences at * *p* < 0.05.

The assay was conducted twice for rigor and relative fluorescence measurements of treatments were compared to the solvent control. In both assays, there was no difference between the solvent control and media control. At 4 h of exposure, MMP differed among experimental groups in the first (F _(29,63)_ = 3.23, *p* < 0.0001) and second (F _(29.63)_ = 2.67 *p* < 0.0006) experiment; however, this was due to the positive control (Figure 3) reducing MMP and there were no differences in the MMP of SH-SY5Y cells following exposure to neither BPS nor BPF following a post hoc correction. Data from both experiments suggest that BPS and BPF do not affect the MMP in SH-SY5Y cells under this exposure regime (Figure 3 and Supplemental Figure S1).

### 3.4. ATPase Activity

**Figure 4:**
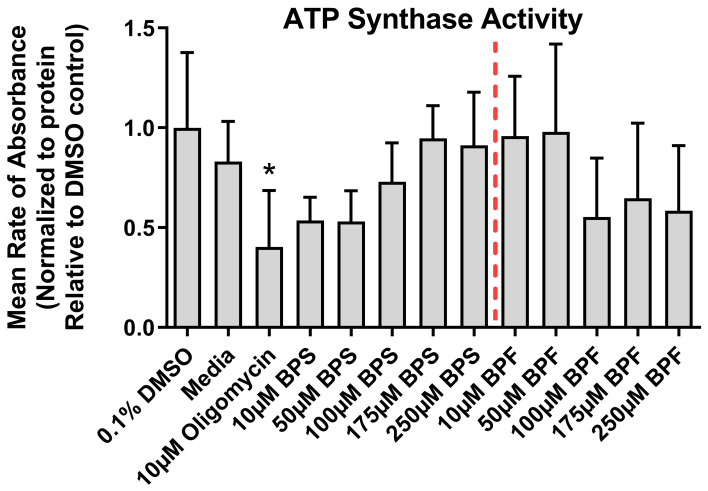
ATP synthase activity following exposure to media, 0.1% DMSO, 10 µM oligomycin, or one concentration of either BPF or BPS at 10, 50, 100, 175, or 250 µM. The column represents mean rate of absorbance ± standard deviation standardized to the DMSO solvent control (One-way ANOVA with a Dunnett’s multiple comparison test, *n* = 4/experimental group). Each treatment was compared to the DMSO control. The red line delineates BPS from BPF. Asterisks indicate significant differences at * *p <* 0.05.

ATPase activity differed significantly among treatments (F _(12,39)_ = 2.1, *p* = 0.037) (Figure 4). However, ATPase activity was only inhibited by the positive control (10 µM oligomycin) and not by any concentration of BPS or BPF. Based on these results, we concluded that ATPase activity is not altered by BPS nor BPF under these experimental conditions.

### 3.5. Reactive Oxygen Species

**Figure 5:**
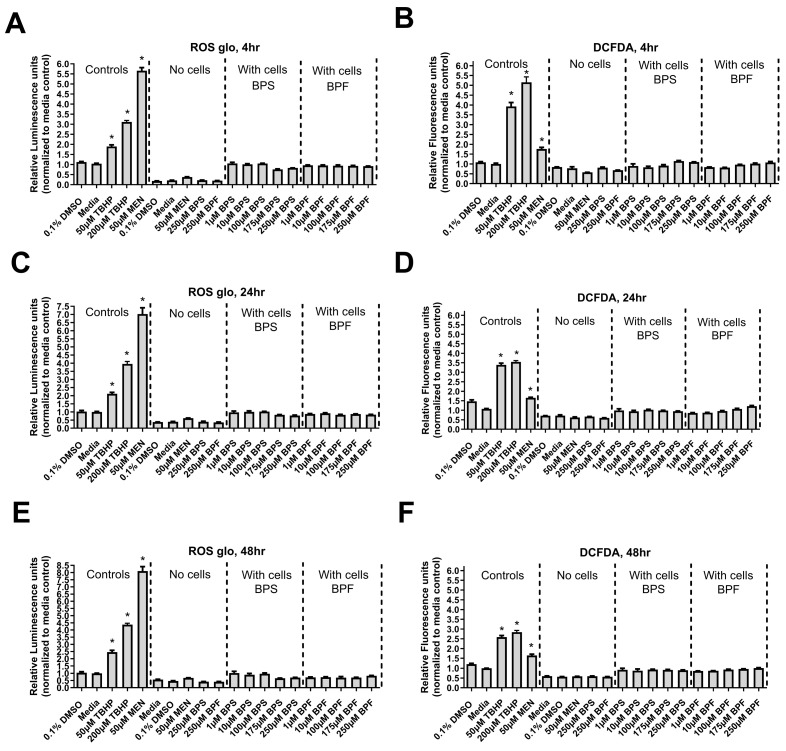
Reactive oxygen species levels in SH-SY5Y cells after exposure to bisphenols. (**A**–**C**) 4, 24, and 48 h ROS-Glo assay and (**D**–**F**) 4, 24, and 48 h DCFDA-based assay following exposure to 0.1% DMSO, media alone, 50 and 200 µM tert-Butyl hydroperoxide (TBHP), 50 µM menadione (MEN), or one concentration of either 1, 10, 100, 175, or 250 µM BPF or BPS. The column represents mean relative luminescence ± standard deviation from the control (One-way ANOVA with a Dunnett’s multiple comparison test, *n* = 4/experimental group). Each treatment was compared to the DMSO control. Asterisks indicate significant differences at * *p <* 0.05.

ROS was measured by two well established methods (Ros-Glo and DCFDA method) in SH-SY5Y cells, following a 4, 24, and 48 h exposure to one of several concentrations spanning 1 to 250 µM. Neither BPS nor BPF induced ROS following 4, 24, and 48 h exposure (Figure 5A–F). However, both positive controls (MEN and TBHT) increased ROS as expected (*p* < 0.05).

### 3.6. Mitochondrial Bioenergetics

A mitochondrial stress test was conducted to measure oxygen consumption rates in cells following a 4 h direct injection exposure to one concentration of either BPS or BPF. We tested concentrations of BPS and BPF that ranged 0.1 nM to 250 µM. We hypothesized that BPS and BPF would alter oxidative consumption rates of SH-SY5Y cells: an indicator of mitochondrial dysfunction. BPS did not affect oxygen consumption rates of SH-SY5Y mitochondria and did not affect oligomycin-induced ATP production nor maximum respiration (Supplemental Figure S2). BPF affected oxygen consumption rates of SH-SY5Y mitochondria but only at the highest concentration tested, and basal respiration was decreased over time following exposure to BPF (Supplemental Figure S3) (*p* < 0.05).

### 3.7. Transcriptome Profiling in SH-SY5Y Cells Following BPS and BPF Exposure

The transcriptomes obtained were overall of a high quality, generating 35.3–47.2 million raw reads per sample (Supplemental Data). Total mapping to the human reference genome was high (92.94–95.91% total mapped and 86.44–89.84% uniquely mapped rates) and fragments per kilobase of transcript per million mapped reads (FPKM) levels were similar across samples.

**Figure 6:**
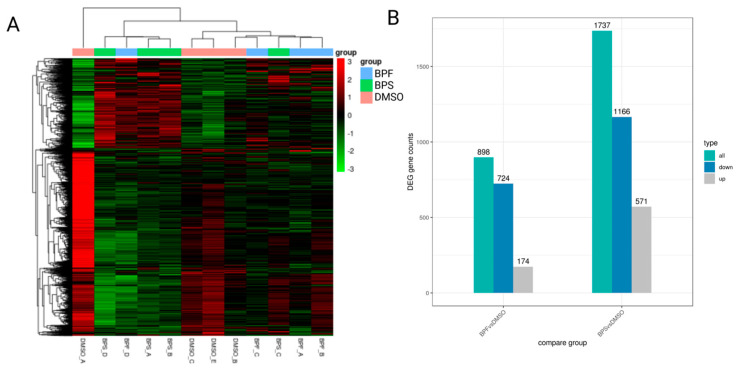
(**A**) Clustering shown as a heatmap. (**B**) Differentially expressed genes in SH-SY5Y cells following exposure to either 0.1 nM BPF or BPS.

Transcriptome patterns, in general, were unique amongst the three experimental groups (control, BPS and BPS) (Figure 6A). The number of differentially expressed genes in SH-SY5Y cells exposed to BPS was 1737 while 898 transcripts were differentially expressed in SH-SY5Y cells exposed to BPF (*p*-value < 0.05) (Figure 6B, Supplementary Data). For BPS, there were 30 transcripts that passed an FDR correction while a single transcript was altered by BPF (*p*-adj < 0.05). Table 1 presents the top 10 expressed candidates in the BPS treatment, while the single transcript that passed an FDR correction in the BPF treatment was uncharacterized. Transcripts most affected by BPS included those related to translation (ribosomes) and metabolism.

**Figure 7:**
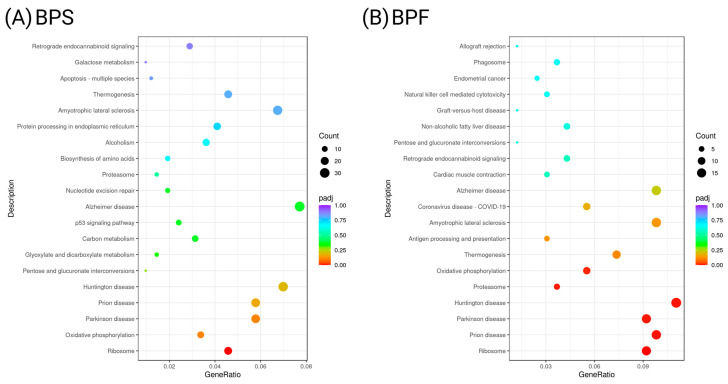
Enrichment of KEGG pathways for (**A**) BPS and (**B**) BPF, depicted as bubble plots where *p*-values are represented by different colors and the numbers of genes are represented by the bubble size.

Enrichment analysis with KEGG pathways is depicted as bubble plots. The enrichment of KEGG pathways was consistent with the cell network enrichment analysis conducted in the Pathway Studio. The sole prominent gene network enriched following BPS exposure was related to ribosomes (Figure 7A). Prominent gene networks that were enriched following BPF exposure were those related to oxidative phosphorylation, proteasomes, Huntington’s disease, Parkinson’s disease, prion disease, and ribosomes (Figure 7B).

The top 10 pathways enriched in each dataset generated from BPS and BPF exposure are presented in Table 2. All GSEA data can be found in the Supplemental Data. In cells treated with BPS, gene sets enriched included Humoral Immunity in Vitiligo, Natural Killer T-Cell Roles in Diabetes Mellitus Type 1, and IL15R triggering NF-kB/NFATC Signaling among others. In cells treated with BPF, gene sets enriched included GPCRs Family activating Expression Targets in Bone, TNF activating NF-kB Expression Targets, and Acute Phase in Atopic Dermatitis, among others. Gene set enrichment analysis also identified common elements between BPS and BPF. These included immune-related signaling pathways (e.g., “CD4+ T-Cell Function Decline in HIV”, “Natural Killer Cell Activation”, and “IL2 Expression Targets”) and insulin-related networks (e.g., “Natural Killer T-Cell Roles in Diabetes Mellitus Type 1”, “Insulin activation of MEF/MYOD Expression Targets”, and “IGF1 activation of ELK/SRF/HIF1A/MYC/SREBF Expression Targets”) (Table 3).

**Figure 8:**
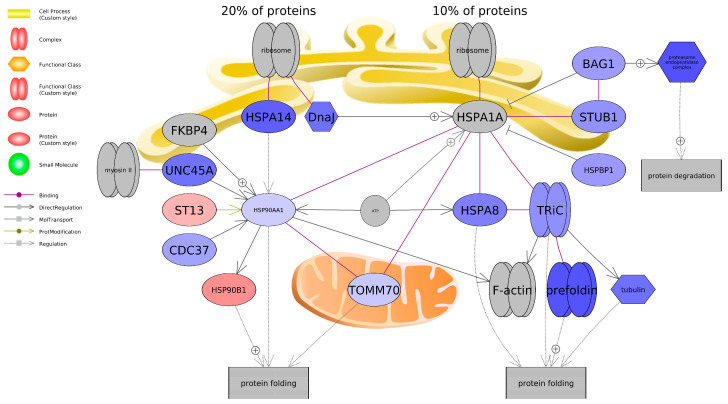
Gene network associated with protein folding in SH-SY5Y cells exposed to BPF. Red indicates upregulation, and blue indicates downregulation of the entity. Fold change, *p*-values, and abbreviations are given in Supplemental Data. The legend indicates type of interactions between entities. For example, each shape is designated a certain biomolecule and the arrows in the pathway indicate relationships among entities like binding, promotor binding, or direct regulation. Plus signs (+) indicate promoting interactions.

**Figure 9:**
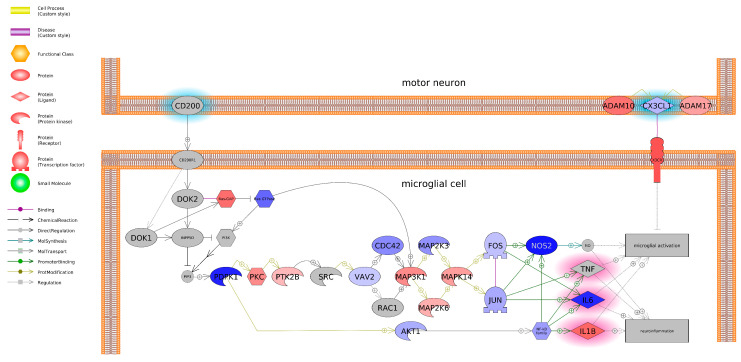
Gene network associated with microglial and motor neuron dysregulation in SH-SY5Y cells exposed to BPF. Red indicates upregulation, and blue indicates downregulation of the entity. Grey indicates the entity was not measured in the experiment. Fold change, *p*-values, and abbreviations are given in Supplemental Data. The legend indicates the type of interactions between entities. For example, each shape is designated a certain biomolecule and the arrows in the pathway indicate relationships among entities like binding, promotor binding, or direct regulation. Plus signs (+) indicate promoting interactions.

Pathway analysis revealed a gene network associated with protein folding in SH-SY5Y cells exposed to BPF (Figure 8) and microglial and motor neuron dysregulation for BPS (Figure 9), as perturbed at the transcriptome level: both processes that underlie neuroinflammation and neurodegenerative diseases. Even though there was no change in mitochondrial bioenergetics nor MMP, there was evidence of perturbed gene networks related to mitochondrial transport (Supplemental Figure S4).

### 3.8. Real-Time PCR Analysis

**Figure 10:**
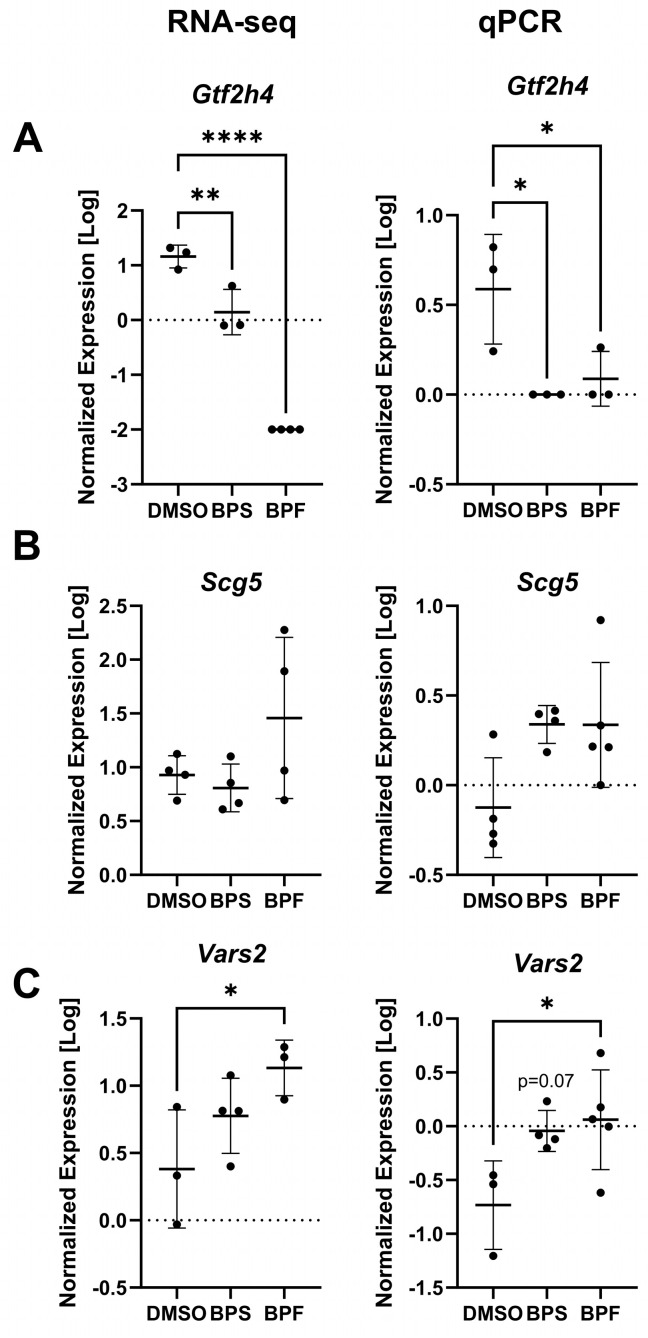
The expression levels (log10 transformed) of (**A**) *Gtf2h4*, (**B**) *Scg5*, and (**C**) *Vars2* in SH-SY5Y cells exposed to either 0.1% DMSO, BPS, or BPF. Each circle is a biological replicate, and the horizontal line indicates the mean (±S.D.). The dotted horizontal line indicates baseline (log = 0) normalized expression. Data analyzed using a One-way ANOVA with a Dunnett’s multiple comparisons test (*n* = 3 to 5 samples/treatment). Asterisks indicate significant differences at * *p* < 0.05, ** *p* < 0.01, **** *p* < 0.0001).

A subset of genes regulated by BPS and BPF were measured to compare to the RNA-seq data (Figure 10). Based on the functional enrichment, genes were related to transcription/translation and measured for correspondence to the RNA-seq data. In general, transcripts followed expression patterns revealed by RNA-seq analysis. These included *Gtf2h4*, qPCR [F _(2, 6)_ = 7.77, *p* = 0.021] and RNA-seq [F _(2, 7)_ = 149.8, *p* < 0.0001]; *Scg5*, qPCR [F _(2, 10)_ = 3.94, *p* = 0.055] and RNA-seq [F _(2, 9)_ = 2.24, *p* = 0.16]; and *Vars*, qPCR [F _(2, 9)_ = 4.41, *p* = 0.046] and RNA-seq [F _(2, 7)_ = 4.21, *p* = 0.063].

## 4. Discussion

Bisphenol A has been restricted or banned in many countries because of the reported detrimental effects on human health associated with its exposure. Accordingly, derivatives (i.e., bisphenol B, E, F, and S) have been formulated to replace the utilization of BPA; however, limited data is available on the impact bisphenol derivatives have on toxicity mechanisms in neuronal cells. Neuronal cells can exhibit a varying degree of sensitivity towards chemical agents in different cell types; thus, it is important to elucidate different mechanisms of bisphenol-induced toxicity in cell types like neurons.

Our data indicated that both BPS and BPF induce cytotoxicity in SH-SY5Y cells, and responses were both time- and concentration-dependent. Our data suggested that BPS was more cytotoxic (>50 µM at 48 h) to SH-SY5Y cells, compared to BPF (>200 µM at 48 h). Other studies also report cytotoxicity for bisphenol replacements in neuronal cells. Liang et al. [40] exposed human embryonic stem cells to varying concentrations of seven different bisphenols (A, AF, B, E, F, S, and Z). During stem cell differentiation, cells were exposed to up to 300 µM for 24 h and a concentration-dependent decrease in cell viability was observed. Interestingly, BPF and BPS were the least cytotoxic to human embryonic stem cells while BPAF was the most cytotoxic compound. In the study, proliferating cells were exposed from 0.001 to 300 µM for 24 h and all bisphenols, except for BPS, reduced cell viability at the highest tested concentration. Additionally, for 16 days during the differentiation to neuron-like cells, cells exposed to 1, 10, or 100 nM of bisphenols decreased neurite length, suggesting that long-term bisphenol exposure can potentially contribute to neurotoxicity.

Other mammalian cells lines have been investigated for neurotoxicity of bisphenol replacements in rats and mice. Gill and Kumara [26] observed neurodevelopmental effects following rat fetal neural stem cells (rNSCs) exposure to 0.05 µM and 100 µM BPA or BPF for seven days following their differentiation into neurons, astrocytes, or oligodendrocytes. BPA and BPF were found to increase cell proliferation and impact differentiation rates of neurons and oligodendrocytes in a dose-dependent manner. Additionally, morphometric analysis revealed that exposure to BPA and BPF reduced the branching of nerve fibers in the neurons, oligodendrocytes, and astrocytes. In another study, Pang et al. [41] observed that BPA, BPS, and BPB induced neurotoxic phenotypes in the hippocampal HT-22 cell line following exposure to 1 nM up to 100 μM for up to seven days. After just 6 h in the study, ROS production significantly increased in the cells following exposure to all compounds. Strong induction of apoptosis and lactate dehydrogenase (LDH) leakage rates was also detected following exposure for 24 and 48 h. These studies prompted us to also investigate oxidative stress as a potential mechanism for neurotoxicity in the SH-SY5Y cells.

In our study, we did not observe any evidence for mitochondrial dysfunction (ROS production, MMP, ATPase activity) in SH-SY5Y cells following BPS and BPF exposure. These data suggest that mitochondria in SH-SY5Y cells may not be a primary target of BPS/BPF-induced toxicity; however, the mitochondria in other cell types may show increased sensitivity to bisphenol exposure. For instance, two other human neuroblastoma cell lines (IMR-32 and SK-N-SH) exposed to BPA, BPS, and BPB (1 nM up to 100 μM) for 24 h [27] were observed to exhibit increased ROS and MDA content. In the study, MMP was reduced and protein levels of Bcl-2 Antagonist/Killer 1 (*bak1*), Bcl-2-Associated X (*bax*), cytochrome c (*cytc*), and cysteinyl aspartate specific proteinase 3 (*caspase-3*) were increased, while protein levels of B-Cell Leukemia/Lymphoma 2 (*bcl2*) were downregulated. Taken together, BPA, BPS, and BPB induced neurotoxic effects (oxidative stress and apoptosis) via the mitochondrial in these neuroblastoma cells. Another study conducted by Meng et al. [42] exposed hippocampal neurons of rats to BPA, BPS, or BPB (1 μM up to 100 μM) for seven days. Exposure over 24 h induced ROS production and increased MDA content; however, SOD activity decreased significantly. After a seven-day exposure to all bisphenols, cell viability decreased, and apoptosis rate increased. Reduction in MMP was also observed following exposure to BPA. In vitro, Huang et al. [43] exposed human granulosa KGN cells to up to 100 µM BPA, BPAF, BPF, or BPS and noted concentration-dependent ROS production. Specifically, 1–100 µM BPA and BPAF, 10 and 100 µM BPF, and 100 µM BPS induced ROS production. Additionally, antioxidant markers (i.e., catalase, superoxide dismutase, glutathione) were decreased depending on the bisphenol analog and oxidative biomacromolecule damage measured by increased levels of 8-hydroxy 2-deoxyguanosine, malondialdehyde, and protein carbonyl were documented with high concentrations of all tested compounds. We hypothesize that SH-SY5Y cells are more resistant to the effects of bisphenols, relative to other neuronal cells lines. Indeed, there is good evidence that neuronal cell lines show varying degrees of sensitivity to chemical toxicants [44] and this may underscore the differences observed across studies.

As oxidative stress was not noted in the SH-SY5Y cells, we conducted a transcriptome analysis to identify other potential mechanisms related to BPS and BPF neurotoxicity. Our transcriptome analysis was conducted at a low concentration, relevant to potential exposures in the central nervous system [23]. The analysis revealed that a higher number of transcripts were responsive to BPS compared to BPF. This may be related to the relative toxicity potential of each compound. In addition, despite high similarity between the two molecules in chemical structure, the bisphenols perturbed unique signaling pathways in neuronal cells. For example, pathways unique to BPS but not BPF include Humoral Immunity in Vitiligo, Treg-Cell Activation in Diabetes Mellitus, and CD80 triggering AP-1 Expression Targets while pathways unique to BPF but not BPS include IgE Receptors activating Targets in Lymphoid System and Blood, TLR9 Expression Targets, and Muscular Dystrophy, Facioscapulohumeral. However, there were several molecular pathways that were shared between BPF and BPS including Eosinophil Activation, IL2 Expression Targets, and TNF activating NF-kB Expression Targets. Other transcriptome studies show that these pathways/processes are also activated by BPA and its analogs [45,46].

Both bisphenols affected pathways such as “PARK2/PINK1/UCHL1 in Young Onset Parkinson’s Disease”, “TNF activation of NF-kB Expression Targets”, and “Androgen Receptor/FKBP5 Signaling”. Kinch et al. [30] exposed embryonic zebrafish to 0.0068 μM BPA and BPS prior to neurogenesis (10–16 hpf), at the onset of neurogenesis (16–24 hpf), and at late neurogenic periods (24–36 hpf). Exposure to BPA and BPS led to a 180% and 240% increase in neurogenesis of the hypothalamus, respectively. Consequently, hyperactive behavior was observed in fish following the neurogenic window exposure. The pathway of BPA-mediated neurogenesis was shown to be associated with androgen receptor mediated upregulation of aromatase; thus, there is evidence that bisphenol replacements can be associated with neurological disorders, such as hyperactivity, and this may perturb early brain development especially during the neurogenic period of development. Several studies also elaborate on the connection of bisphenol exposure with neurodegenerative diseases (i.e., Parkinson’s disease, Alzheimer’s disease, and amyotrophic lateral sclerosis) and neurodevelopmental disorders (i.e., autism and hyperactivity) [25]. Sukjamnong et al. [47] observed that prenatal rat exposure to 5000 μg/kg BPA disrupts the transcriptome profiles of Alzheimer’s-related and neuroinflammatory genes in the hippocampus of offspring. Levels of tumor necrosis factor (*tnf*)*,* nuclear factor-κB (NF-κB) protein, and its Alzheimer’s Disease related target gene (beta-secretase 1, *bace1*) increased. Thus, bisphenol exposure has been linked to neurodegenerative diseases.

Based on the literature and databases that detail gene-protein interactions between entities, pathway analysis revealed that BPF impacted genes related to protein folding and BPS impacted genes related to microglia and motor neuron dysregulation. Most transcripts identified as being associated with a protein folding were downregulated and included Heat Shock Protein Family A Member 8 (HSPA8) and Member 14 (HSPA14), Heat Shock Protein 90 Alpha Family A Member 1 (HSP90AA1), and Translocase of Outer Mitochondrial Membrane 70 (TOMM70), among others. On the other hand, BPS upregulated both A Disintegrin and Metalloproteinase Domain 10 (ADAM10) and Domain 17 (ADAM17). Consequently, these genes promote microglial activation, which can contribute to neuroinflammation. Intriguingly, we found that both BPS and BPF affected pathways related to neuroinflammation, such as interleukin 2 (IL2), NF-κB, and TNF, and the two bisphenols are reported to impact similar pathways of in vivo and in vitro models; however, studies focus more on in vivo rodent models. Takahashi et al. [48] exposed pregnant mice to 20 and 200 μg/kg/day BPA over the course of their pregnancy and analyzed embryonic offspring brains where, compared to the controls, levels of inflammatory genes and signaling molecules were altered, including microglial markers, inflammatory factors (TNFα and interleukin 4), and a neurotrophic factor (IGF-1). Similar neuronal inflammatory markers have also been reported by other studies. Akintunde et al. [49] exposed rats to 100 mg/kg BPA for two weeks and noted elevated neuronal interleukin-1β (IL-1β), TNFα, acetylcholinesterase (AChE), and monoamine oxidase-A (MAO-A) activity in the brain. Additionally, phosphodiesterase-5′ activity was enhanced, in which large increases may promote neuroinflammation [50]. In another study, Luo et al. [51] noted a 2.58- and 2.35-fold increase in TNF-α and interleukin-6 (IL-6), respectively, in the prefrontal cortex of F1 juvenile mice after mothers were treated with 50 mg/kg BPA for two weeks. Regarding in vitro models, BPA treatment (10 nM–100 μM) increased hypothalamic pro-opiomelanocortin (POMC)-expressing mice cells and cultures, which was due to induced neuroinflammatory pathways (i.e., IKK-β/NFκB) [52]. Taken together, neuroinflammatory pathways appear to be altered with BPS and BPF exposure.

## 5. Conclusions

In summary, we evaluated the relative toxicity of BPS and BPF in a widely used neuronal cell line for degenerative disease research. We noted that in general, BPS exerted higher toxicity to the cells, inducing cytotoxicity and reducing cell viability at lower concentrations than BPF. We tested the hypothesis that the mitochondria of neuronal cells were a major target of BPS and BPF toxicity; however, negative effects on mitochondria were not readily detected based upon mitochondrial membrane potential, assessment of ROS levels, and mitochondrial bioenergetics. This suggests that mitochondria may not be a direct target of BPS or BPF neurotoxicity, especially at low acute levels of exposure. Nevertheless, chronic low concentration exposures may still act to stress neurons and impair mitochondrial function.

Limitations of this study must be considered. As SH-SY5Y cells are tumor-derived, they are more resistant to cellular stress; thus, they are less sensitive to mitochondrial dysfunction. Consequently, this may explain why we did not observe mitochondrial dysfunction following BPF/S exposure, whereas other studies utilizing different neuronal cell types have reported such outcomes. Additional research using a range of neuronal models and in vivo systems is warranted to better understand these mechanisms. For example, future research utilizing additional neuronal cell models would be beneficial in understanding the neurotoxicity of BPF/S; nevertheless, SH-SY5Y cells are a valuable neurotoxicology cell model for the initial screening of cytotoxic effects, which can be terminally differentiated into a non-proliferative cell type with retinoic acid (in fact, this is a treatment for this type of cancer). In addition, cell lines are susceptible to genetic drift, and passage number is a crucial parameter. For these experiments, we ensured cells remained at a low passage number (<13). Lastly, one future step is to validate some of the key pathways (e.g., IL15R activating NF-kB/NFATC) using targeted pharmacological inhibitors or siRNA knockdown approaches to strengthen transcriptome data. Proteomics approaches can also be carried out to complement the transcriptomics data.

We identified several molecular pathways that may underscore BPS-induced neurotoxicity at relevant levels, including IL15R activating NF-kB/NFATC signaling and CD8 activating NF-kB expression targets. Similarly, we identified several molecular pathways that may be involved in BPF-induced neurotoxicity, including TLR9 expression targets and TNF activation of NF-kB expression targets. RNA-seq analysis revealed that, despite high conservation between the molecules in the chemical structure, the bisphenol compounds perturb unique signaling pathways in neuronal cells. Thus, neuroinflammation may be a more prevalent mechanism of neurotoxicity for bisphenols relative to mitochondrial dysfunction but this hypothesis must be rigorously tested. Taken together, acute exposures to bisphenol replacements may not have notable consequences on the phenotype of SH-SY5Y neuronal cells; however, prolonged exposures may yield greater phenotypic responses and toxicity.

## Figures and Tables

**Table 1:** Top regulated DEGs in human SH-SY5Y cells following exposure to BPS (ranked by *p*-value). The table presents gene abbreviations, gene names, log2 fold change, *p*-adjusted, and *p*-values. Log2FC refers to fold change relative to control.

Gene Name	Full Name	log2FC	*p*-Adj	*p*-Value
RPS2P55	Ribosomal Protein S2 Pseudogene 55	−1.455	0.001	4.4149 × 10^−8^
MT-TT	Mitochondrially Encoded TRNA-Thr (ACN)	1.587	0.006	6.6467 × 10^−7^
HLA-C	Major Histocompatibility Complex, Class I, C	−2.063	0.006	1.4419 × 10^−6^
AC016596.2	Ribosomal protein L41	−1.929	0.006	1.7420 × 10^−6^
RPL35P5	Ribosomal Protein L35 Pseudogene 5	−2.069	0.006	1.7752 × 10^−6^
AC010343.1	Ribosomal protein S8 pseudogene	−1.794	0.006	2.1002 × 10^−6^
RPS10P3	Ribosomal Protein S10 Pseudogene 3	−2.368	0.007	2.7400 × 10^−6^
Unknown	Unknown	−2.907	0.007	3.1888 × 10^−6^
DUSP8	Dual Specificity Phosphatase 8	−1.488	0.007	3.4916 × 10^−6^
AC011005.1	Unknown	−2.424	0.007	3.9371 × 10^−6^

**Table 2:** Top pathways enriched in each transcriptome dataset following exposure to BPS or BPF. The table reports the name of the pathway, number (#) of pathway entities, expanded number (#) of entities, number (#) of measured entities, median change, the normalized enrichment score, and the *p*-value. Data are presented in Supplemental Data.

Chemical	Name	# of Entities	Expanded # of Entities	# of Measured Entities	Median Change	Normalized Score	*p*-Value
BPS	Humoral Immunity in Vitiligo	94	161	63	−1.06	1.71	0.0017
	Natural Killer T-Cell Roles in Diabetes Mellitus Type 1	82	132	56	1.03	1.78	0.0018
	T-Cell Maturation (Hypothesis)	77	436	52	1.04	1.66	0.0018
	IL2 Expression Targets	97	138	61	−1.05	1.77	0.0018
	Atopic Dermatitis Onset	72	348	51	−1.06	1.69	0.0018
	Treg-Cell Activation in Diabetes Mellitus	72	121	57	1.03	1.67	0.0018
	CD8 Activation of NF-kB Expression Targets	44	63	30	1.05	1.73	0.0019
	IL15R Activation of NF-kB/NFATC Signaling	15	22	10	−1.21	1.80	0.0019
	RAS/RAF/MAPK Signaling Activation by Blocking of Tumor Suppressors	47	427	27	−1.06	1.79	0.0019
	CD80 Activation of AP-1 Expression Targets	28	32	14	1.07	1.80	0.0019
BPF	Dectin-2 (CLEC6A)/Mincle (CLEC4E)/BDCA2 (CLEC4C) Signaling	23	33	11	−1.02	1.86	0.0019
	IgE Receptors Activation of Targets in Lymphoid System and Blood	25	70	6	−1.38	1.73	0.0020
	GPCRs Family Activation of Expression Targets in Bone	15	17	6	1.33	−1.64	0.0021
	S/G2 Phase Transition	49	247	132	−1.13	1.51	0.0032
	TNF Activation of NF-kB Expression Targets	127	136	62	−1.05	1.72	0.0033
	TLR9 Expression Targets	42	47	24	1.03	1.82	0.0035
	CNR Activation of Expression Targets in Nerve Tissue	35	89	14	1.01	1.73	0.0036
	Acute Phase in Atopic Dermatitis	53	82	22	1.04	1.69	0.0038
	Muscular Dystrophy, Facioscapulohumeral	32	343	10	1.21	1.62	0.0039
	Aminoglycosides and Cisplatin-Induced Ototoxicity (Mouse Model)	25	237	9	−1.13	1.70	0.0040

**Table 3:** Top overlapping pathways following exposure to BPS or BPF. The table reports the name of the pathway, number (#) of measured entities, median change, and the *p*-value. Data are presented in Supplemental Data.

Overlapping Pathways	# of Measured Entities BPF	Median Change BPF	*p*-Value PBF	# of Measured Entities BPS	Median Change BPS	*p*-Value PBS
CD4+ T-Cell Function Decline in HIV	56	−1.006	0.005	57	1.006	0.004
Natural Killer T-Cell Roles in Diabetes Mellitus Type 1	57	−1.008	0.047	56	1.030	0.002
Eosinophil Activation	58	−1.047	0.007	59	−1.111	0.026
IL2 Expression Targets	61	−1.058	0.014	61	−1.047	0.002
TNF Activation of NF-kB Expression Targets	62	−1.048	0.003	63	−1.043	0.005
CD8+ T-Cell Response in Celiac Disease	65	−1.076	0.016	64	−1.096	0.004
Natural Killer Cell Activation	65	−1.076	0.032	64	−1.079	0.012
Protein Folding	69	−1.224	0.021	71	−1.264	0.035
IGF1 Activation of ELK/SRF/HIF1A/MYC/SREBF Expression Targets	74	−1.107	0.037	74	−1.101	0.036
Insulin Activation of MEF/MYOD Expression Targets	77	−1.056	0.029	79	−1.125	0.046

## Data Availability

All transcriptome data is available through the NCBI Gene Expression Omnibus at Accession (GSE217951).

## Supplementary Materials

The following supporting information can be downloaded at: https://www.mdpi.com/article/10.3390/toxics13090772/s1. Supplemental Methods, Figures, and Data. Data also available in Zenodo (doi: 10.5281/zenodo.16187837). Figure S1: Mitochondrial membrane potential (MMP) of SH-SY5Y cells treated with 0.1% DMSO, media, 4 and 8 μM carbonyl cyanide-4-phenylhydrazone (FCCP), or one concentration of either BPF or BPS at 0.001 µM up to 250 µM after 4 h. This is the second experiment conducted with the bisphenols. Data are expressed as relative mean intensity units ± standard deviation standardized to the solvent control (One-way ANOVA followed by a Dunnett’s multiple comparison test, *n* = 3/treatment/experiment). All treatments were compared to the DMSO control. Asterisks indicate significant differences at * *p* < 0.05, ** *p* < 0.01, *** *p* < 0.001, **** *p* < 0.0001). Figure S2: Normalized oxygen consumption rate for differentiated SH-SY5Y cells after exposure to bisphenol S. Data are graphed as mean ± SEM (*n* = 5). The control is the solvent control (0.1% DMSO). Different letters denote significant difference from DMSO control (One-way ANOVA followed by a Dunnett multiple comparison test, *n* = 4, significance determined at *p* < 0.05). Figure S3: Normalized oxygen consumption rate for differentiated SH-SY5Y cells after exposure to bisphenol F. Data are graphed as mean ± SEM (*n* = 5). The control is the solvent control (0.1% DMSO). Different letters denote significant difference from DMSO control (One-way ANOVA followed by a Dunnett multiple comparison test, *n* = 4, significance determined at *p* < 0.05). Figure S4: Gene network for protein folding. Red indicates that the transcript is increased relative to control, and green means that the transcript or inferred complex is downregulated compared to control. Fold change data are provided in Supplemental Data for each transcript in the network. Abbreviations are provided in Supplemental Data (For interpretation of the references to color in this figure legend, the reader is referred to the web version of this article).

## Abbreviations

The following abbreviations are used in this manuscript:

AChE	Acetylcholinesterase
ADAM10	A Disintegrin and Metalloproteinase Domain 10
ADAM17	A Disintegrin and Metalloproteinase Domain 17
AM/Rot	Antimycin/Rotenone
BAK1	Bcl-2 Antagonist/Killer 1
BAX	Bcl-2-Associated X
BACE1	Beta-secretase 1
BLC-2	B-Cell Leukemia/Lymphoma 2
BPA	Bisphenol A
BPF	Bisphenol F
BPS	Bisphenol S
Casp3/7	Caspase 3/7
Caspase3	Cysteinyl aspartate specific proteinase 3
CYT C	Cytochrome c
DCFDA	2′,7′-dichlorofluorescein diacetate
DIG	Digitonin
DMSO	Dimethyl sulfoxide
ER	Estrogen receptor
FCCP	Carbonyl cyanide-4-phenylhydrazone
FPKM	Fragments per kilobase of transcript per million
GEO	Gene Expression Omnibus
GO	Gene Ontology
GPCR	G-protein coupled receptors
GSEA	Gene set enrichment analysis
HSPA8	Heat Shock Protein Family A Member 8
HSPA14	Heat Shock Protein Family A Member 14
HSP90AA1	Heat Shock Protein 90 Alpha Family A Member 1
IL-1β	Interleukin-1β
IL2	Interleukin 2
LDH	Lactate dehydrogenase
MAO-A	Monoamine oxidase-A
MDA	Malondialdehyde
MEN	Menadione
MMP	Mitochondrial membrane potential
NF-κB	Nuclear factor-κB
OM	Oligomycin
POMC	Pro-opiomelanocortin
RA	Retinoic acid
rNSCs	Rat fetal neural stem cells
ROS	Reactive oxygen species
TBHP	Tert-Butyl hydroperoxide
TNF	Tumor necrosis factor
TOMM70	Translocase of Outer Mitochondrial Membrane 70
