# Supplementary material for: Exposure to Bisphenol S and Bisphenol F Alters Gene Networks Related to Protein Translation and Neuroinflammation in SH-SY5Y Human Neuroblastoma Cells
Source: Toxics. 2025 Sep 12;13(9):772. doi: 10.3390/toxics13090772 (PMC12474282; doi:10.3390/toxics13090772)
Supplement: Supplementary file 1 [file toxics-13-00772-s001.zip › Supplemental Methods.pdf]

Supplemental Methods

Exposure to Bisphenol S and Bisphenol F Alters Gene Networks Related to Protein Translation and Neuroinflammation in SH-SY5Y Human Neuroblastoma Cells

Andrea P. Guzman †, Christina L. Sanchez †, Emma Ivantsova, Jacqueline Watkins, Sara E. Sutton, Christopher L. Souders II and Christopher J. Martyniuk *

Center for Environmental and Human Toxicology, Department of Physiological Sciences, College of Veterinary Medicine, UF Genetics Institute, Interdisciplinary Program in Biomedical Sciences Neuroscience, University of Florida, Gainesville, FL 32611, USA; apguzman525@gmail.com (A.G.); chrissylaurasanchez@gmail.com (C.L.S.); eivantsova@ufl.edu (E.I.); jmecalwatkins@ufl.edu (J.W.); sarasutton@ufl.edu (S.S.); ksouders@ufl.edu (C.L.S.II)

* Correspondence: cmartyn@ufl.edu

† These authors contributed equally to this work.

2.1 *Chemicals*

PESTANAL®, analytical standards for both BPF (4,4′-Methylenediphenol) (CAS Number 620-92-8) and BPS (4,4′-Sulfonyldiphenol) (CAS Number 80-09-1) were purchased from Sigma-Aldrich (St. Louis, MO, USA). Nominal stock solutions of BPS and BPF were prepared by dilution in dimethyl sulfoxide (DMSO) (CAS no. 67-68-5, purity ≥99.9%, Sigma-Aldrich, USA). Oligomycin A (≥99%, CAS Number 579-13-5), carbonyl cyanide-4-phenylhydrazone (≥98%, CAS Number: 370-86-5), and antimycin A (≥95%, CAS Number 1397-94-0) were also purchased from Sigma for mitochondrial assays.

2.2 SH-SY5Y culturing, differentiation, and chemical exposures

Cells were purchased from ATCC® CRL 2266™ and validated by the company through STR profiling. Differentiated SH-SY5Y neuroblastoma were used for all experiments because they produce more catecholamines (dopamine and epinephrine) versus undifferentiated cells and are more neuronal in phenotype. We employed retinoic acid (RA) (10 μM) for 6 days for SH-SY5Y differentiation as per established methods (Kovalevich and Langford, 2013; Xie et al., 2010). For differentiation, media consisted

of DMEM:F12 supplemented with 1% FBS, antibiotic/antimycotic solution, and 10 μM retinoic acid. Media changes were performed every 48 hours for a total of 6 days prior to chemical exposures which occurred on Day 7. We have observed that SH-SY5Y cells treated for 6 days and more with 10 μM RA exhibit neurite outgrowths and stain strongly for the dopaminergic phenotype, based on tyrosine hydroxylase staining.

Cells were first maintained in DMEM:F12 media supplemented with 10% (v/v) heat-inactivated Fetal Bovine Serum (hiFBS) and 1% (v/v) antibiotic-antimycotic (100×; Invitrogen, USA) at 5% CO_2 at 37 °C. Cells were passaged from a T25 flask by washing with 5 mL Hanks Balanced Salt Solution with 0.25% trypsinization and differentiation occurred using the following protocol. Cells were seeded on Day 1, and 10 μM RA was added on Day 0, Day 2, Day 4, Day 6. Thus, cells were treated with the chemicals on Day 7. Cells were treated in either wells or flasks, which were protected from light with foil and incubated in a 37°C CO_2 incubator for the desired period. Differentiated SH-SY5Y cells were maintained in DMEM:F12 with L-glutamine media supplemented with 1% (v/v) fetal bovine serum, and 1% Antibiotic-Antimycotic solution (Gibco) in a humidified atmosphere of 5% CO_2 at 37 °C (Korecka et al., 2013).

2.3 Cell viability assay

The CellTiter-Glo® Luminescent Cell Viability Assay (Promega) was used to determine the proportion of viable SH-SY5Y cells in the culture, which is based on the quantitation of ATP, indicating the presence of metabolically active cells. Briefly, SH-SY5Y cells were collected and differentiated in an incubator at 37 °C in white 96-well culture plates at 5% CO_2 before treatment. Cells were exposed to media only, 0.1% DMSO solvent control, positive control (20 μM Antimycin/Rotenone), or one dose of either BPF or BPS at 0.1, 0.5, 1, 5, 10, 50, 100, 150, 200, or 250 μM (n=4). After cells were treated, CellTiter-Glo reagent was added to each well, and plates were incubated at room temperature for 10 minutes. The 96-well plate was placed on an orbital shaker platform at 200 rpm for 2 minutes. The luminescence was recorded at using a Synergy™ 4 Hybrid Microplate Reader (BioTek).

2.4 Caspase activity assay

SH-SY5Y cells were collected as outlined above and were pelleted by centrifugation (100 g for 6 min at 4°C) and then gently washed (3x) in ice-cold 1X phosphate-buffered saline (PBS). Cells were

seeded at n=3 (wells/dose) after being differentiated for 6 days with retinoic acid. Cells were treated with one concentration of either BFS or BPF over a dose range of 0.1-250 µM. Briefly, 100 µL of media containing ~10,000 cells were seeded in each well of a 96-well plate. Cells were also treated with different toxicants like 30 µg/mL digitonin (DIG), and 1, 10, and 100 µM Antimycin A and Rotenone at each time point. These chemicals acted as positive controls for the assay to induce cytotoxicity and caspase activity. Each time point was conducted in different plates. At either 4, 24, and 48 hours, 100 µL of Caspase-Glo 3/7 reagent (Promega) was added to each well. After 30 seconds of orbital shaking at 400 rpm, the plate was incubated for 1.5 hours at room temperature. Luminescence was determined using a Synergy™ 4 Hybrid Microplate Reader (BioTek). Background fluorescence of cells was considered and controlled for each assay (background corrected).

2.5 Mitochondrial membrane potential

Mitochondrial membrane potential (MMP) was measured using the Mitochondrial Membrane Potential Kit (Sigma-Aldrich). Briefly, cells were first differentiated with RA in black 96-well plates. After 6 days, cells were exposed to media only, 0.1% DMSO solvent control, positive control (4 and 8 µM FCCP), or one dose of either BPF or BPS at 0.001, 0.01, 0.1, 0.5, 1, 5, 10, 50, 100, 150, 175, 200, and 250 µM (n=3). Following treatment, 50 µL of JC10 dye solution “A” was added to each well using a multichannel pipette and the cells were incubated at 37 °C for 30 minutes before 50 µL of lysis solution “B” was added to each well and fluorescence excitation/emission intensities of 540/590 nm (red) and 490/525 nm (green) were measured using a Synergy™ 4 Hybrid Microplate Reader (BioTek). Graphs represent the proportion of red/green fluorescence intensities per each well. The experiment was conducted twice, independently.

2.6. ATPase activity

SH-SY5Y cells were differentiated in 24-well plates for 6 days with RA, then exposed in quadruplicate for 24 hours to one concentration of 10, 50, 100, 150, 175, or 250 µM BPS or BPF, or the positive control (10 µM oligomycin). Following the exposure, SH-SY5Y cells were transferred into 1.7 mL tubes and frozen at -80°C, thawed, and resuspended in solution one

media, supplied in the assay kit (ATPase Assay Kit, ab234055). Samples were then homogenized, and a BCA protein assay was performed to normalize the data. Detergent was then added at 1/10th dilution, and cells were centrifuged for 20 minutes at 16,000 RPM. Following this, 50 µL of each sample was then transferred to a 96-well plate coated with monoclonal antibodies specific to ATP synthase and incubated at room temperature for 3 hours. Wells were then emptied and washed twice with 300 µL of solution one media and emptied before 40 µL of lipid mix was added. After a 45 minute incubation, 200 µL of reagent mix was added, bubbles were avoided, and absorbance at 340 was read on a BioTek Synergy H1 Spectrophotometer each minute for an hour using a kinetic assay program.

2.7 Mitochondrial bioenergetics in differentiated SH-SY5Y cells

SH-SY5Y were seeded following an established procedure for preventing edge effects in adherent cell lines (Lundholt et al., 2003). In brief, SH-SY5Y cells were seeded at density of 25,000 cells per 100 µL of differentiation media (DMEM-F12 +1% heat inactivated fetal bovine serum +1% antibiotics/antimycotics) in a Seahorse XFe24 cell culture microplate and allowed to attach for 6-8 hours before the addition of 150 µL of supplemental media. Background wells (A1, B4, C3, and D6) were seeded with 100 µL of differentiation media lacking cells. The following day, a 10 µM concentration of retinoic acid (RA) was obtained via a 1:1 dilution (i.e., adding 250 µL of differentiation media containing 20 µM RA to the 250 µL already present in each well). Media changes were performed with the lights turned off to minimize a photolysis of chemicals and were conducted before plates were returned to the 37 °C incubator. Cell culture microplates were wrapped in aluminum foil to further prevent exposure to light. Approximately 70% media changes of 10 µM RA in differentiation media were performed every two days. After 6 days of differentiation, cells were exposed to the experimental conditions via another 1:1 dilution following a pseudo-randomized design scheme using a direct injection routine.

Under the direct injection scheme, the cell plate was washed on the sixth day of differentiation with seahorse incubation media directly, and allowed to incubate in a 37 °C non- CO_2 incubator for 1 hour, before being analyzed using a Seahorse XFe24 flux analyzer following the standard

mitochondrial stress test regimen with final concentrations of 0.5 μM oligomycin, 12 μM FCCP, and 1 μM Antimycin A. There was an additional injection of 10X concentrations of the experimental chemicals following basal respiration cycles. Cells were exposed to media only, 0.1% DMSO, or one final concentration of either 0.1 nM, 0.1 μM, 100 μM, or 250 μM BPS with the injection. Cycles were 2 minutes of mixing, 1 minute wait, and 3 minutes of measurements. For the direct injection scheme, there were three measurements for basal OCR, followed by injection of the chemical for six cycles/measurements, three measurements following oligomycin injection, three measurements following FCCP injection, and three measurements at the end after addition of antimycin A. Final OCR was normalized to total protein within each well according to a BCA protein assay [562nm (Absorbance)]

2.8. Intracellular reactive oxygen species in differentiated SH-SY5Y cells

Reactive oxygen species (ROS) production was measured using the ROS-Glo™ H_2O_2 Assay (Promega, Cat #G8820). Briefly, 10,000 SH-SY5Y cells were seeded per well of a white 96-well plate and differentiated using 10 μM retinoic acid in 200 μL (changed every 48 hours). On the sixth day, media was reduced to 70 μL and cells were exposed to one dose of BPF or BPS ranging from 1 μM up to 250 μM for 4 hours by adding 10 μL of 10X stocks together with 20 μL H_2O_2 substrate solution. The plate was then returned to a 37°C incubator for 4 hours, after which 100 μL of reconstituted ROS-Glo™ Detection Solution was added to each well. The plate was then incubated at room temperature for 20 minutes before luminescence was measured using a Synergy™ 4 Hybrid Microplate Reader (Biotek).

SH-SY5Y were seeded at 20,000 cells/100 μL in each well of a black 96-well plate (excluding four no cell control wells). The following day, cells were differentiated by diluting the 100 μL with attached cells with 100 μL of 20μM retinoic acid (RA) to produce a 10 μM RA solution. Two and four days later, a 50% media change was performed to replace the 10 μM RA with fresh equimolar solution. On day 6, 100 μL of media was removed and 100 μL of 2X chemical stocks were added with a multichannel, using a second 96-well plate filled with 200 μL of 2X chemical solution. Immediately following this, 22μL of 10X DCFDA solution (500 μM) was added with a multichannel to each well. The plate was mixed at 400 rpm for 30 seconds via orbital shaker before being returned to a 37ºC CO2 incubator for 45 minutes. Bubbles were then popped before the plate was read on a Synergy H2 Plate Reader at 485/535, top read, auto gain

using a Kinetic assay design, measuring fluorescence every 5 minutes for 6 hours. The time was noted when chemical and DCFDA were initially added, as well as when the kinetic assay began recording. During data analysis, overflow measurements were redefined as 100,000, since this is the upper limit of detection for this plate reader.

2.9 RNA-sequencing

Cells were exposed in T75 flasks by removing all the media and replacing it with 8 mL of differentiation media (DMEM:F12 containing 1% FBS, 1% antibiotic/antimycotic solution, and 10 μM retinoic acid) every 48 hours. On the sixth day, cells were exposed to media, 0.1 nM BPS, or 0.1 nM BPF (n=4-5) for 48 hours. Media was then removed, and the remaining cells were added into TRIzol™ Reagent and stored at -80°C for further processing. Total RNA was extracted using TRIzol™ Reagent (Thermo Fisher Scientific, Waltham, MA USA) as per the manufacturer's instruction. RNA concentration was determined using the Qubit® 2.0 Fluorometer (ThermoFisher, Grand Island, NY, USA). RNA quality was assessed using the Agilent 2100 Bioanalyzer (Agilent Technologies, Inc.). A total of 12 RNA samples were deemed to be high quality for RNA-seq library construction (RINs>7).

Libraries and sequencing were conducted by Novogene (Novogene Corporation, Beijing, China). Briefly, enrichment of mRNA proceeded using the NEBNext Poly(A) mRNA Magnetic Isolation module (New England Biolabs, catalog # E7490). RNA library construction followed instructions outlined in the NEBNext® Ultra™ II Directional RNA Library Prep Kit for Illumina® (New England Biolabs, catalog #E7760). Individually prepared libraries were pooled by equimolar concentrations and sequenced using a NovoSeq 6000 instrument (150 bp paired end reads) (Illumina Inc., CA, USA). Raw data (raw reads) of FASTQ format were first processed through fastp. In this step, clean data (clean reads) were obtained from raw data by removing reads containing adapter and poly-N sequences and reads with low quality. At the same time, Q20, Q30 and GC content of the clean data were determined. All downstream analyses were based on clean data with high quality.

The reference genome (homo_sapiens_grch38_p12_gca_000001405_27) and gene model annotation files were downloaded from the Ensembl genome website browser directly. HISAT2 software was used for alignment of RNA-seq data. Mapping information from all samples was combined as input

into StringTie assembler. The assembled transfrags were then compared to the reference transcripts to determine if they were sufficiently different to be considered novel. This analysis can identify novel genes, identify novel exons of known genes, and is used to optimize the start and end information of known transcripts. Once mapped, gene expression levels were determined by the abundance of transcripts (count of sequencing) that mapped to the human genome or exon. FPKM (Fragments Per Kilobase of transcript sequence per millions base pairs sequenced) was used to estimate gene expression levels, considering both sequencing depth and gene length on counting of fragments. To identify differentially expressed genes, read counts were first normalized and FDR values for each transcript generated, based on multiple hypothesis testing.

2.10 Bioinformatics of pathways

As a first-level analysis, enrichment analysis of the differential expressed genes was conducted for each concentration of BPF and BPS using the Gene Ontology (GO). The Kyoto Encyclopedia of Genes and Genomes (KEGG) annotates genes to pathway level. In a second level analysis, we performed Gene Set Enrichment Analysis (GSEA) to determine whether the prior gene set is significantly different between two biological states (e.g., phenotype). Gene set enrichment analysis (GSEA) and subnetwork enrichment analysis (SNEA) were conducted in Pathway Studio 12.0 (Elsevier, Amsterdam, Netherlands). Gene set enrichment analysis proceeded with 1000 permutations to generate the distributions. Pathway Studio conducted statistical enrichment based upon ontologies and curated pathways. A two-sample nonparametric Kolmogorov–Smirnov test was used to compare the cumulative distributions of two data sets (networks) for differences.

SNEA is designed around networks of common regulators of expression using known relationships (i.e., expression, binding, etc.) derived for experimental data and literature, which are focused on gene hubs. A distribution of expression values is calculated by a permutation algorithm to obtain a “background” distribution followed by a statistical comparison between the sub-network data (query data) and the background distribution using a Mann–Whitney U-Test. For both GSEA and SNEA, a P-value is generated to indicate whether a process is statistically enriched in the query data set, relative to what is expected by random chance based upon the background distribution. Enrichment P-value for a

gene seed was set at P < 0.05. GSEA and SNEA lists are provided in the Supplementary Data. All raw and processed transcriptome data are available via the NCBI Gene Expression Omnibus (GEO) database (GSE217951, release date April 2024).

2.11 Real-time PCR analysis

Real-time PCR followed our established protocols using TRIzol® Reagent (Life Technologies, Carlsbad, CA, USA) (Souders II et al., 2021). Following extraction using TRIzol® Reagent, RNA integrity was determined using the RNA 6000 nano kit and 2100 Bioanalyzer (Agilent Technologies, Santa Clara, CA, USA). Samples with RNA integrity values greater than seven were used for downstream analyses. Genomic DNA was removed using the TURBO DNA free™ Kit as per the manufacturer's instructions (ThermoFisher Scientific). The cDNA step was conducted using 500 ng RNA and the iScript™ Select cDNA Synthesis Kit (Bio-Rad, CA, USA). Three "no reverse transcriptase (NRT)" controls were prepared in the same fashion, except water was used instead of enzyme. The T100™ Thermal Cycler (BioRad, USA) was used to cycle temperatures needed to generate cDNA as per the manufacturer's instruction. The CFX Connect System (BioRad, USA) was used to perform a quantitative polymerase chain reaction (RT-qPCR) with SSo-Fast™ EvaGreen® Supermix Kit (BioRad, Hercules, CA, USA). Samples were run in duplicate and followed RT-qPCR cycling parameters described by us (Souders II et al., 2021).

The primers used in this study were obtained from the published literature or designed using NCBI Primer. Based on the functional enrichment, genes related to transcription/translation were measured. These included general transcription factor IIH subunit 4 (Gtf2h4) *(BT007321.1) [F' TATTGGACCGATTGTATGGGCA, R' AGCCCTGTACTTTCCTCCTGA]*
Secretogranin V (Scg5) (NG_051230.1) [F' CTGTCCTGTTGGAAAAACAGCAG, R' GACACTCCTCCGCTTTCGTC], and Valyl-tRNA synthetase 2 (Vars2) (BC113605) [F' ACAGCCCCCGATATGTTGAG, R' GGCCTGATATTCTGGTTTGAAGA]. Expression data were normalized to *Gapdh* [F' TGCACCACCAACTGCTTAGC, R' GGCATGGACTGTGGTCATGAG] (Cicinnati et al., 2008) using CFX Manager™ software (v3.1) (baseline subtracted) and the Cq method was employed.

2.12 Statistical analysis

All statistical analyses were conducted in GraphPad Prism (La Jolla, CA, USA, version 9.4). Differences for cell viability data, caspase activation, MMP, ATP synthase levels, and ROS were analyzed using a One-way ANOVA, followed by a Dunnett's multiple comparison test to the DMSO solvent control. Relative mRNA levels were first log transformed (log10) and analyzed with ANOVA, followed by a Dunnett's test. Data are presented as mean ± S.D unless otherwise stated in figure caption. Significance of difference was considered when $p < 0.05$ for all endpoints.
