# Supplementary material for: Exposure to Bisphenol S and Bisphenol F Alters Gene Networks Related to Protein Translation and Neuroinflammation in SH-SY5Y Human Neuroblastoma Cells
Source: Toxics. 2025 Sep 12;13(9):772. doi: 10.3390/toxics13090772 (PMC12474282; doi:10.3390/toxics13090772)
Supplement: Supplementary file 1 [file toxics-13-00772-s001.zip › Supplemental Figures.pdf]

Supplemental Figures

Exposure to Bisphenol S and Bisphenol F Alters Gene Networks Related to Protein Translation and Neuroinflammation in SH-SY5Y Human Neuroblastoma Cells

Andrea P. Guzman †, Christina L. Sanchez †, Emma Ivantsova, Jacqueline Watkins, Sara E. Sutton, Christopher L. Souders II and Christopher J. Martyniuk *

Center for Environmental and Human Toxicology, Department of Physiological Sciences, College of Veterinary Medicine, UF Genetics Institute, Interdisciplinary Program in Biomedical Sciences Neuroscience, University of Florida, Gainesville, FL 32611, USA; apguzman525@gmail.com (A.G.); chrissylaurasanchez@gmail.com (C.L.S.); eivantsova@ufl.edu (E.I.); jmecalwatkins@ufl.edu (J.W.); sarasutton@ufl.edu (S.S.); ksouders@ufl.edu (C.L.S.II)

* Correspondence: cmartyn@ufl.edu

† These authors contributed equally to this work.

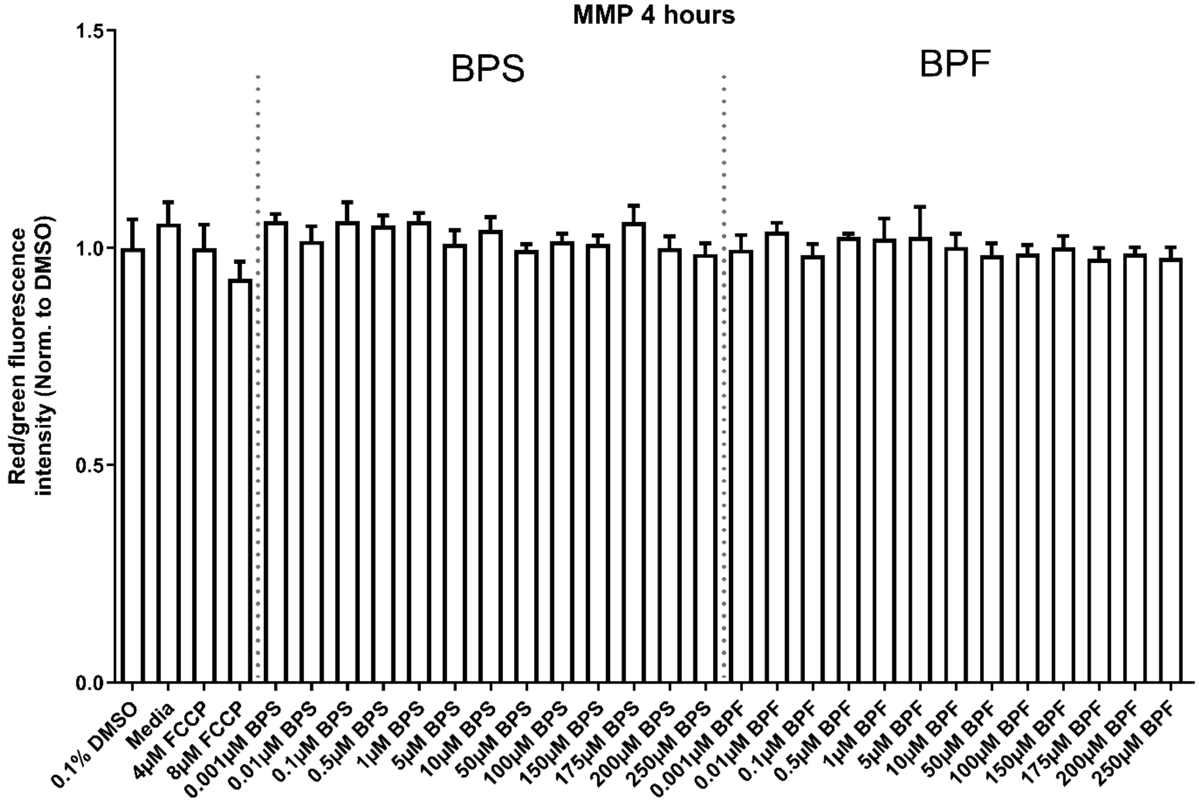

Figure S1. Mitochondrial membrane potential (MMP) of SH-SY5Y cells treated with 0.1% DMSO, media, 4 and 8 µM carbonyl cyanide-4-phenylhydrazone (FCCP), or one concentration of either BPF or BPS at 0.001 µM up to 250 µM after 4 hours. This is the second experiment conducted with the bisphenols. Data are expressed as relative mean intensity units ± standard deviation standardized to the solvent control (One-way ANOVA followed by a Dunnett's multiple comparison test, n=3/treatment/experiment). All treatments were compared to the DMSO control. Asterisks indicate significant differences at $^{*}p < 0.05$, $^{**}p<0.01$, $^{***}p<0.001$, $^{****}p<0.0001$).

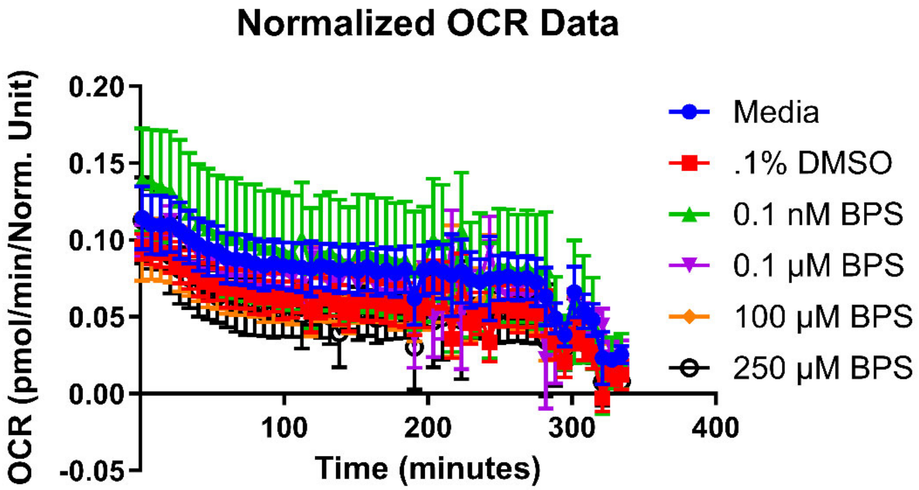

Figure S2. Normalized oxygen consumption rate for differentiated SH-SY5Y cells after exposure to bisphenol S. Data are graphed as mean ± SEM (n=5). The control is the solvent control (0.1 % DMSO). Different letters denote significant difference from DMSO control (One-way ANOVA followed by a Dunnett multiple comparison test, n=4, significance determined at $p<0.05$).

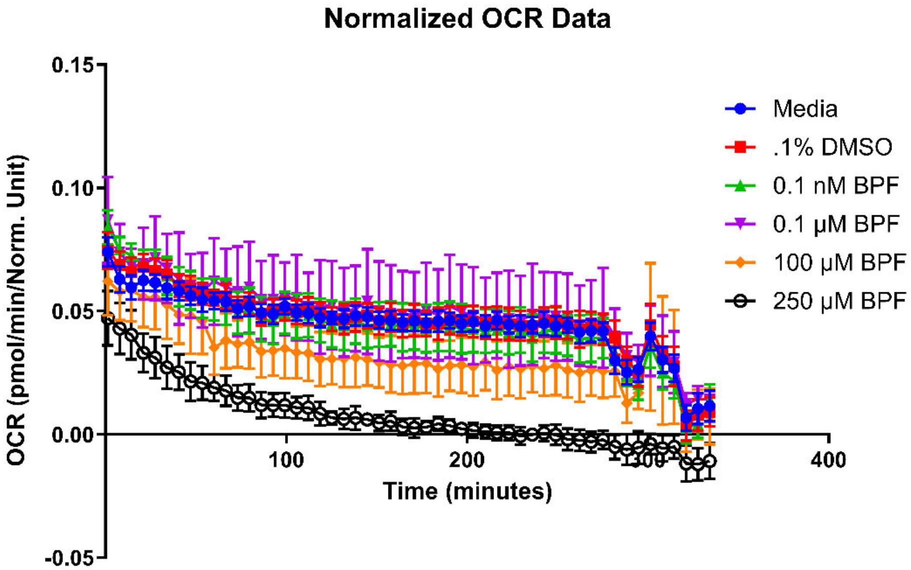

Figure S3. Normalized oxygen consumption rate for differentiated SH-SY5Y cells after exposure to bisphenol F. Data are graphed as mean ± SEM (n=5). The control is the solvent control (0.1 % DMSO). Different letters denote significant difference from DMSO control (One-way ANOVA followed by a Dunnett multiple comparison test, n=4, significance determined at $p<0.05$).

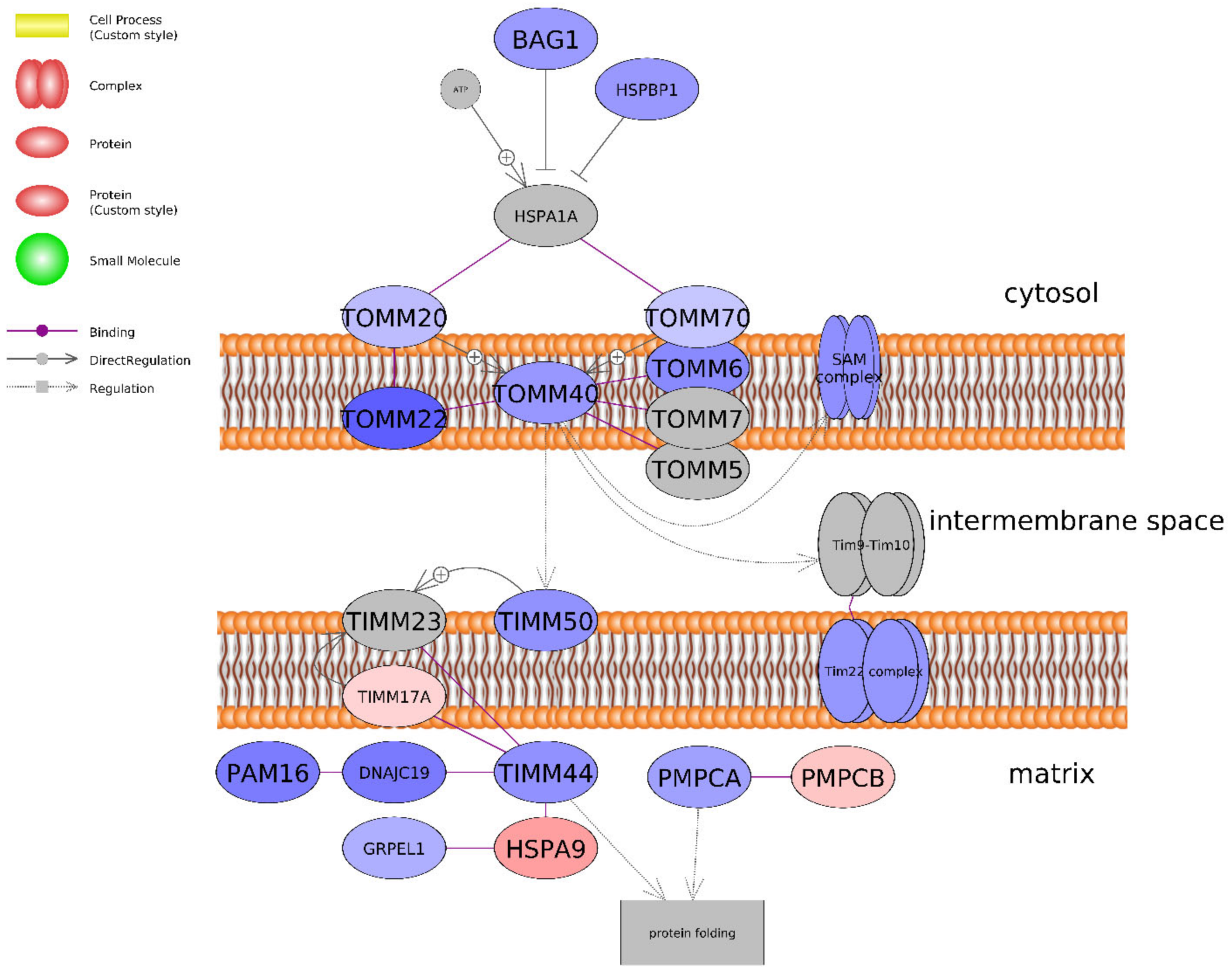

Figure S4. Gene network for protein folding. Red indicates that the transcript is increased relative to control, and green means that the transcript or inferred complex is downregulated compared to control. Fold change data are provided in Supplemental Data for each transcript in the network. Abbreviations are provided in Supplemental Data (For interpretation of the references to color in this figure legend, the reader is referred to the web version of this article).
